# Critical Role of HAX-1 in Promoting Avian Influenza Virus Replication in Lung Epithelial Cells

**DOI:** 10.1155/2018/3586132

**Published:** 2018-01-16

**Authors:** Xue Li, Bingqian Qu, Ganlin He, Carol J. Cardona, Yongchun Song, Zheng Xing

**Affiliations:** ^1^Medical School and the Jiangsu Provincial Key Laboratory of Molecular Medicine, Nanjing University, Nanjing, China; ^2^Department of Veterinary Biomedical Sciences, College of Veterinary Medicine, University of Minnesota Twin Cities, St. Paul, MN 55108, USA; ^3^College of Biological Sciences, Nanjing University, Nanjing, China

## Abstract

The PB1-F2 protein of influenza A virus has been considered a virulence factor, but its function in inducing apoptosis may be of disadvantage to viral replication. Host mechanisms to regulate PB1-F2-induced apoptosis remain unknown. We generated a PB1-F2-deficient avian influenza virus (AIV) H9N2 and found that the mutant virus replicated less efficiently in human lung epithelial cells. The PB1-F2-deficient virus produced less apoptotic cells, indicating that PB1-F2 of the H9N2 virus promotes apoptosis, occurring at the early stage of infection, in the lung epithelial cells. To understand how host cells regulate PB1-F2-induced apoptosis, we explored to identify cellular proteins interacting with PB1-F2 and found that HCLS1-associated protein X-1 (HAX-1), located mainly in the mitochondria as an apoptotic inhibitor, interacted with PB1-F2. Increased procaspase-9 activations, induced by PB1-F2, could be suppressed by HAX-1. In HAX-1 knockdown A549 cells, the replication of AIV H9N2 was suppressed in parallel to the activation of caspase-3 activation, which increased at the early stage of infection. We hypothesize that HAX-1 promotes AIV replication by interacting with PB1-F2, resulting in the suppression of apoptosis, prolonged cell survival, and enhancement of viral replication. Our data suggest that HAX-1 may be a promoting factor for AIV H9N2 replication through desensitizing PB1-F2 from its apoptotic induction in human lung epithelial cells.

## 1. Introduction

H9N2 virus is a low pathogenic avian influenza virus and has remained a concern to not only the poultry industry but also human health. Studies have found that human bronchial epithelium is susceptible to H9N2 [1], which is able to cross the species barrier [2] and has infected humans since 1999. H9N2 virus has been proposed to be the internal gene donor for genesis of high pathogenic AIV H5N1 and recently emerging AIV H7N9 via genetic reassortment when H9N2 virus cocirculated in poultry markets in contact with migratory birds carrying other subtypes of AIV [3, 4].

PB1-F2 protein was originally identified through screening antigenic peptides recognized by CD8^+^ T lymphocytes [5], encoded in the PB1 gene at an alternative reading frame. About 96% of the AIV strains have a full-length PB1-F2, while human H1N1 viruses either possess truncated 57 amino acids or lack the protein completely [6]. However, PB1-F2 is dispensable in pandemic (2009) H1N1 virus which was demonstrated in a study that, if PB1-F2 was restored in the form of full-length via reverse genetics, there was no effect on viral replication and pathogenesis in mice [7]. In addition, PB1-F2 is of no importance in the pathogenesis of seasonal H1N1 virus-infected ferrets [8]. Except for these roles in influenza monoinfection, PB1-F2 primes and promotes more pulmonary immunopathology in the superinfection leading to secondary bacterial pneumonia, especially caused by Gram-positive pathogens [9, 10]. It suggests that PB1-F2 may function differently in infection by different AIV subtypes.

Mitochondrial targeting sequence was identified at the whole C-terminus of H1N1 (PR8) PB1-F2 [5], while H5N1 PB1-F2 with a distinct C-terminal does not target or partially targets the mitochondria [11]. C-terminus residues forming *α*-helix were regarded as minimally required (Lys73, Arg75) for mitochondrial targeting sequence [12]. Mapping studies characterized other residues located at the C-terminus (Ser66 in H5N1; Pro62, His75, Gln79, and Ser82 in H3N2; Ile68, Leu69, and Val70 in H1N1) that are strongly associated with proinflammatory and pathogenic effects induced by PB1-F2 [13–16]. These studies suggested a correlation between mitochondrial localization and its function. However, the role of PB1-F2 N-terminus is poorly understood, although two short and weak *α*-helixes were proposed [17].

Mitochondrial location and membrane-binding activity of PB1-F2 support it as an apoptotic and proinflammation inducer as well as an enhancer in secondary bacterial superinfections. This apoptotic sensitization was originally observed mainly in immune cells [5]. Next, PB1-F2 was found to localize mainly in the mitochondria of A549 cells, capable of inducing apoptosis by interacting with the inner mitochondrial membrane adenine nucleotide translocator 3 (ANT3) and the outer mitochondrial membrane voltage-dependent anion channel 1 (VDAC1), leading to higher mitochondrial membrane permeability and cytochrome c release [18].

We have limited knowledge on the effect of H9N2 PB1-F2 induced-apoptosis in viral replication, virulence, and pathogenesis. Studies have shown that apoptosis in influenza virus infection could be suppressed in various cell types [19–22]. Our previous data indicated that increased apoptosis impaired influenza virus replication and the viral nonstructural protein NS1 promoted viral replication in part through its suppression of the Fas-FasL apoptotic signaling during an H9N2 infection [22]. A recent report is showing that highly pathogenic H5N1 virus delayed apoptosis in human bronchial and alveolar epithelial cells to prolong the duration of viral replication, which contributes to H5N1 pathogenesis [20]. The question remains, however, whether PB1-F2-induced apoptosis could be regulated through some mechanisms that involve PB1-F2 by itself during the infection. In this study, we report that HCLS1-associated protein X-1 (HAX-1) is directly associated with PB1-F2 of an H9N2 AIV, and the association contributes to suppressed apoptosis induced by PB1-F2 in human lung epithelial cells. We propose that direct protein-protein interaction of HAX-1 with PB1-F2 may be critical in PB1-F2-induced apoptosis and viral replication in H9N2 virus-infected human lung epithelial cells.

## 2. Materials and Methods

### 2.1. Cells and Reagents

Mardin-Darby canine kidney (MDCK) cells, human embryonic kidney (HEK) 293T cells, and human lung epithelial carcinoma A549 cells were cultured in this study with DMEM medium (Invitrogen, Carlsbad, CA), 10% fetal bovine serum (HyClone, Logan, UT), 1 mM sodium pyruvate (Amresco, Solon, OH), and 1% antibiotic-antimycotic solutions (Invitrogen) at 37°C in an incubator with constant 5% CO_2_. Transfection reagent TransIT-LT1 was purchased from Mirus Bio (Madison, WI). Primary antibodies for cytochrome c (sc-7159), HA tag (sc-805), *β*-actin (sc-47778), and HAX-1 (sc-166845) were obtained from Santa Cruz Biotechnology (Santa Cruz, CA), and Flag-tag M2 monoclonal antibody was purchased from Sigma-Aldrich (St. Louis, MO). Anti-cleaved caspase-9 (number 9501) antibody, anti-cleaved caspase-3 (number 9664) antibody, and horseradish peroxidase (HRP) or alkaline phosphatase- (AP-) conjugated anti-rabbit IgG were obtained from Cell Signaling Technology (Danvers, MA). HRP-conjugated anti-mouse IgG was purchased from Sunshine Biotechnology (Nanjing, China).

### 2.2. Viruses and Mutants Generated by Reverse Genetics

The influenza A virus strain A/pheasant/CA/2373/1998 (H9N2) is a low pathogenic AIV isolated from a pheasant as described previously [23]. We propagated this H9N2 virus by inoculating 10-day-old pathogen-free chicken embryonated eggs. After incubation at 37°C for 48 hours (hrs), allantoic fluid was harvested and titrated in MDCK cells by a plaque assay. All eight genomes were cloned in a pDP2000 vector [24].

**Figure 1:**
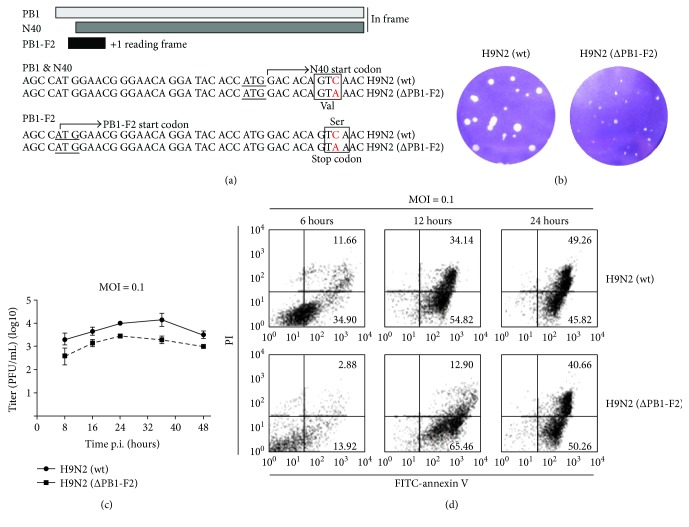
PB1-F2 was critical to influenza virus replication and sensitized human lung epithelial cells for apoptosis. (a) Generation of a PB1-F2-deficient H9N2 virus mutant, H9N2 (ΔPB1-F2). A premature stop codon (TAA) was introduced to replace a serine (TCA) in wild-type virus, H9N2 (wt), without altering a valine in the PB1 and N40 coding sequences. (b) Reduced size of viral plaques in the absence of PB1-F2. MDCK cells were inoculated with the H9N2 (wt) or H9N2 (ΔPB1-F2) viruses (on the right), and a standard plaque assay was performed. (c) Decreased viral titers of H9N2 (ΔPB1-F2) replicating in human lung epithelial cells A549. The titration of viral titers was duplicated, and mean ± SD values were shown (Student's *t-*test, *p* < 0.05). (d) Early apoptosis induced by PB1-F2. A549 cells were infected with H9N2 (wt) or H9N2 (ΔPB1-F2) and trypsinized at 6, 12, and 24 hrs post infection to obtain single-cell suspensions, which were subsequently stained with FITC-annexin V and propidium iodide (PI) before being analyzed with flow cytometry.

To generate a PB1-F2-deficient virus, a single cytosine-to-adenine nucleotide substitution was introduced into the PB1-expressing plasmid pDP2000 to convert a serine (encoded by TCA) at the amino acid position 12 of PB1-F2 to a stop codon (TAA). This nucleotide substitution within +1 open reading frame did not alter the corresponding valine in the frame of PB1 and PB1-N40, and thereby, those two proteins were not changed (Figure 1(a)). A reverse genetics approach was used to generate the mutant H9N2 virus with eight pDP2000-based plasmids [22, 24] designated as H9N2 (ΔPB1-F2). Wild-type H9N2 (wt) virions were produced by cotransfection of the eight original plasmids. Both H9N2 (wt) and H9N2 (ΔPB1-F2) viruses were further propagated in chicken embryonated eggs, and the resultant allantoic fluid was harvested prior to viral titration and aliquots were stored at −80°C. Before infection experiments, RNA was extracted from a virus stock and all viral genomes were sequenced to confirm again that only the single-nucleotide mutation was introduced (data not shown).

### 2.3. Plaque Assay

MDCK cells were seeded in 6-well plates and cultured at 37°C overnight until an 80% confluent monolayer was formed. Allantoic fluid from virally inoculated embryonated eggs or culture media from infected cells were 10-fold serially diluted in DMEM medium before addition to the wells. After viral attachment for 1 hour, the viral inocula were discarded and the cells rinsed with PBS twice before 2 ml of 1% plaque-grade agarose in Opti-MEM (Gibco, Grand Island, NY) containing 2 *μ*g tolylsulfonyl phenylalanyl chloromethyl ketone (TPCK) trypsin (Sigma-Aldrich) were added. After 48- to 72-hour incubation, absolute numbers of forming plaques in each dilution were counted to calculate infectious virus titers (plaque forming units, PFU/ml). Agarose overlays were carefully removed from plates, and cells were stained with crystal violet.

### 2.4. Screening PB1-F2 Interacting Proteins

The Matchmaker Gold Yeast Two-Hybrid system (Clontech Laboratories, Mountain View, CA) was applied to screen host proteins interacting with the PB1-F2 protein of the H9N2 AIV. Full-length PB1-F2 complementary DNA (cDNA) was cloned into pGBKT7 downstream of the *Gal4* gene in fusion as bait, and a pGAD-based human lymphoid cDNA library (Clontech) was used for screening AH109 yeast cells according to the manufacturer's protocol. After initial screening, over a dozen positive candidates were identified. Interactions were further confirmed in subsequent cotransformation with individually purified cDNA clones and the PB1-F2 cDNA in yeast, which resulted in positive clones for further analyses including DNA sequencing. Next, N-terminal or C-terminal PB1-F2 was similarly cloned into pGBKT7 that was used for the identification of PB1-F2 and HAX-1 interaction.

### 2.5. Western Blot Analysis and Coimmunoprecipitation (Co-IP)

Transfected or infected cells were lysed in lysis buffer (50 mM Tris, 150 mM NaCl) with 1% NP-40, 2 mM PMSF, 1 mM Na_3_VO_4_, 2 mM NaF, and 1 *μ*g/ml aprotinin (Sigma-Aldrich). Lysates were centrifuged at 400g for 5 minutes, and cytosolic fractions of cell lysates were transferred, heat denatured, and subjected to SDS-PAGE and proteins were subsequently blotted to Immuno-Blot PVDF membrane (Millipore, Billerica, MA) for western blot analysis. Membranes were blocked with 5% blotting-grade milk and incubated with primary antibodies (rabbit anti-cleaved caspase-9 or anti-cleaved caspase-3) at 4°C overnight. After four washes with TBS containing 0.05% Tween 20 (TBST), the membranes were incubated with AP- or HRP-conjugated secondary antibodies for 90 minutes at room temperature before further washes. For detection with AP-conjugated secondary antibodies, NBT/BCIP substrate (Invitrogen) was used for signal development on the membrane; for detection with HRP-conjugated secondary antibodies, ECL-Plus Western Blotting Detection reagents (Beyotime, Hangzhou, China) were used and the membranes were exposed to the G:BOX Chemi systems (Syngene, MD).

To overexpress PB1-F2 and HAX-1 in mammalian cells, full-length PB1-F2 was amplified from pDP2000-PB1 with an N-terminal Flag tag and subcloned in the pRK5 backbone. HAX-1 was amplified from A549 cDNA with an N-terminal HA tag and inserted into the pRK5 vector. For Co-IP analysis, HEK293T cells were cotransfected with the same amounts of pRK5-Flag-PB1-F2 and pRK5-HA-HAX-1. After 16 hrs, the cells were completely lysed as described above. 200 *μ*l of the quantified lysates was incubated with 1 *μ*g of mouse anti-Flag M2 (Sigma-Aldrich, F1804) or anti-HA tag (C29F4) antibody (Cell Signaling Technology, number 3956) at 4°C for overnight incubation. 20 *μ*l of protein A/G-agarose beads was added into lysates and incubated for 2 hrs at 4°C to precipitate the antigen-antibody complex. The beads were washed with lysis buffer 3 times at 4°C, and then SDS-loading buffer was added and the immunoprecipitates were denatured for western blot analysis.

### 2.6. Colocalization of PB1-F2 and HAX-1 in Mitochondria

To visualize subcellular localization of PB1-F2 and HAX-1, we constructed pcDNA3.1-Zeo(−)-HA-HAX1, pcDNA3.1-Zeo(+)-Flag-PB1-F2, and pcDNA3.1-Zeo(+)-HA-PB1-F2. A549 cells grown on coverslips in 12-well plates were transfected with 1 *μ*g plasmids of pcDNA3.1-HA-HAX-1 and pcDNA3.1-Flag-PB1-F2. Twenty-four hrs later, Mitotracker (Thermo Fisher Scientific, M7512) was added onto cells at a final concentration of 0.2 *μ*M. After forty-minute incubation at 37°C, cells were fixed with 4% PFA and then permeabilized with 0.25% Triton X-100. The primary antibodies were purchased from Cell Signaling Technology (HA-tag (C29F4) rabbit mAb) and Sigma-Aldrich (monoclonal anti-Flag M2 mouse F1804). The secondary antibodies were anti-Mouse-Alexa Fluor 488 (A-11001) and anti-Rabbit-Alexa Fluor 546 (A-11010) from Thermo Fisher Scientific. After immunofluorescence staining, cells were arranged to confocal (Leica Camera AG) scanning.

### 2.7. shRNA Knockdown of HAX-1

Lentiviral vectors expressing shRNAs, specifically targeting *HAX1* gene, were applied to obtain HAX-1 stable knockdown cell lines. We chose two shRNAs targeting distinct sites in the mRNA of the human *HAX-1* gene, and glycerol stocks of the constructs were ordered from GIPZ lentiviral shRNA library via Thermo Fisher Scientific (Rockford, IL). HAX-1 shRNA sequence information is V2LHS_65286 and V3LHS_343976. To obtain infectious lentiviruses, we cotransfected TLA293 producing cells (Thermo Fisher Scientific) with pGIPZ plasmids containing shRNA sequences and packaging plasmid mix using an Arrest-In transfection reagent (Thermo Fisher Scientific) following the manufacturer's procedures. After 48 hrs post transfection, cultural medium containing lentiviruses was harvested and used to infect A549 cells in 6-well plates. The medium was replaced 48 hrs later; then 1 *μ*g/ml of puromycin was added for the selection of shRNA-expressing cells for one week. The A549 cells were monitored under a Nikon fluorescence microscope daily until all the cells turned green, which relies on the coexpressed TurboGFP signals in the same cassette. Knockdown efficiency in puromycin-selected stable A549 cells was measured by quantitative real-time PCR using HAX-1-specific primers as shown in Section 2.10. Clones with the highest knockdown efficiency were propagated and maintained in 0.8 *μ*g/ml of puromycin for subsequent functional experiments.

### 2.8. Caspase-9 Activity Analysis

A549 cells were transfected with pRK5-HA-HAX-1, pRK5-Flag-PB1-F2, or both. Sixteen hrs post transfection, cells were lysed and total protein was quantified by BCA assay for the normalization of inputs before enzymatic activities were measured [25], which was the approach advised by the reagent suppliers as well (personal communication). Caspase-9 activity was measured by using a caspase-9 activity assay kit (Desenbio, Beijing, China). Briefly, the catalytic activity of caspase-9 from cell lysates was determined by incubating the lysates with a caspase-9 peptide substrate Leu-Glu-His-Asp-p-nitroanilide (LEHD-pNA) at 37°C for 2 hrs. After colorimetric development, absorption values at the wavelength of 405 nm were read. Data were shown as fold change compared to the mock-transfected or uninfected groups. Each panel was performed in triplicate experiments independently.

### 2.9. Flow Cytometric Analysis of Apoptosis

A549 cells were infected with H9N2 (wt) or H9N2 (ΔPB1-F2), respectively, at the same multiplicity of infection (MOI) of 0.1. At various time points, the culture medium was discarded and cells were washed twice with cold PBS prior to trypsinization. One million cells were resuspended in 100 *μ*l of binding buffer; then 10 *μ*l of FITC-conjugated annexin V and 10 *μ*l of propidium iodide (PI) (Invitrogen) were added, and staining reaction was on the ice for 15 mins in the dark. The cells were washed three times with PBS, followed by flow cytometric analysis with a cell sorter (BD FACSAria, Franklin Lakes, NJ).

### 2.10. Quantitative Real-Time PCR

Total RNA from HAX-1_wt_ and HAX-1_kd_ A549 cells was extracted with an RNA simple kit (Tiangen, Beijing, China). 1 *μ*g of total RNA was used for reverse transcription using the Primescript RT reagent kit (Takara, Shiga, Japan). Real-time PCR was performed with 1 *μ*l of the cDNA in a total volume of 10 *μ*l with SYBR Premix Ex Taq II (Takara) following the manufacturer's protocol (HAX-1 primers: sense 5′-agtaacccgacacgaagcag-3′ and antisense 5′-ggaaccaacgtcccaggaat-3′). Relative gene expression levels were standardized by an internal glyceraldehyde-3-phosphate dehydrogenase (GAPDH) control, and fold change of HAX-1 gene expression levels was calculated following the formula 2^(ΔCt of HAX-1 − ΔCt of GAPDH)^ as described previously [22, 26].

### 2.11. Statistical Analysis

Two-tailed Student's *t*-test was used to analyze the data for statistics. A difference with a confidence of 95% or greater (*p* ≤ 0.05) was considered statistically significant.

## 3. Results

### 3.1. PB1-F2 Was Critical to Viral Replication and Affected H9N2-Induced Apoptosis in Human Lung Epithelial Cells

To evaluate the role of viral PB1-F2 in epithelial cells during AIV infection, we generated a PB1-F2-deficient H9N2 virus with a reverse genetics approach. A stop codon was introduced by replacing the nucleotide cytosine at position 142 with an adenine in the PB1-F2 reading frame (Figure 1(a)). The amino acid alteration resulted in an early translational termination and a truncated PB1-F2 without impeding the expression of PB1 and its in-frame truncated N40. The mutant virus was designated as H9N2(ΔPB1-F2). H9N2(ΔPB1-F2) formed smaller viral plaques in size than H9N2(wt) did in the monolayers of MDCK cells (Figure 1(b)). It indicated that PB1-F2 might be critical to viral replication in MDCK cells. Infectious viral plaque assay showed that the titers of H9N2(wt) were higher than those of H9N2(*Δ*PB1-F2), with a significant difference up to nearly 10-fold at 36 hrs p.i. (Figure 1(c)).

To examine if PB1-F2 induced apoptosis in epithelial cells, A549 cells were infected with either H9N2 (ΔPB1-F2) or H9N2 (wt) viruses and subjected to annexin V/PI staining at indicated time points for flow cytometry analysis. Compared to H9N2 (wt) infection, there were less dead cells (annexin V^+^PI^+^) detected in the H9N2-infected group (ΔPB1-F2) at 6 and 12 hrs p.i. However, there was no difference in apoptotic cells between the two groups at the later stage (24 hrs p.i.) (Figure 1(d)). The results indicated that PB1-F2 promoted viral replication. On the other hand, PB1-F2 appeared to also sensitize the cells for apoptosis, occurring at the early stage of infection, which may play a negative role for viral replication by shortening survival of host cells. PB1-F2 promotes H9N2 viral replication unlikely through its apoptotic sensitization. There could be an unknown mechanism that may regulate apoptosis induced by PB1-F2 during the infection.

### 3.2. PB1-F2 Interacts with HAX-1

We postulated that PB1-F2 may be regulated by a cellular component, which affects its induction of apoptosis. Thus, we decided to screen a human cDNA library with the yeast two-hybrid approach for proteins interacting with PB1-F2. A dozen proteins were identified as listed in Table 1, including SIVA1 apoptosis-inducing factor, retinoic acid-induced 2, and ubiquitously expressed prefoldin-like chaperone. HCLS1-associated protein X-1 (HAX-1) was among those identified (Table 1).

**Figure 2:**
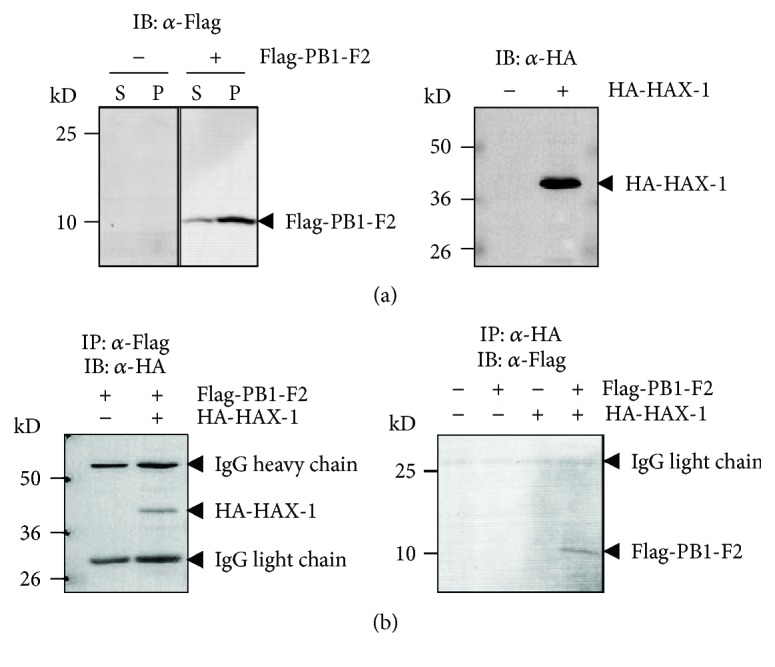
PB1-F2 interacted with HAX-1. HEK293T cells were transfected with pRK5-Flag-PB1-F2 or pRK5-HA-HAX-1. The NP-40 soluble fraction (S) and insoluble pellet (P) of the cell lysates were subjected to western blot analysis with either anti-Flag or anti-HA antibodies to detect expression of PB1-F2 or HAX-1. (a) Only the soluble fraction was tested for HAX-1 expression. (b) Coimmunoprecipitation of PB1-F2 and HAX-1. HEK293T cells were mock transfected or transfected with pRK5-Flag-PB1-F2 and pRK5-HA-HAX-1. The cell lysates were prepared and incubated with either anti-Flag (left) or anti-HA (right) antibody and immunoprecipitated, which were subjected to western blot analysis with either anti-HA (left) or anti-Flag antibody (right).

To confirm the interaction between HAX-1 and PB1-F2 in the cells, HEK293T cells were cotransfected with pRK5-Flag-PB1-F2 and pRK5-HA-HAX-1, followed by coimmunoprecipitation (co-IP) and western blot assay. Both HA-tagged HAX-1 and Flag-tagged PB1-F2 were expressed in transfected cells; especially, PB1-F2 could be detectable in both soluble (S) and insoluble (P) fractions of the cell lysates (Figures 2(a)). In the cell lysates, HA-HAX-1 and Flag-PB1-F2 could be coimmunoprecipitated, detected in both antibodies, indicating that HAX-1 was associated with PB1-F2 in cotransfected cells (Figure 2(b)).

### 3.3. Decreased Viral Replication in HAX-1 Knockdown A549 Cells

**Figure 3:**
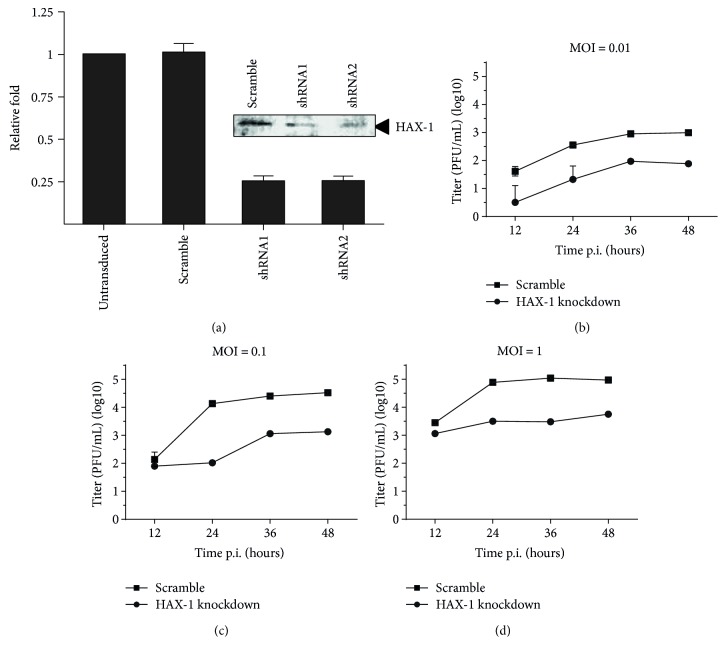
HAX-1 promoted H9N2 influenza virus replication in the lung epithelial cells. (a) Generation of human lung epithelial cell lines with decreased HAX-1 expression through shRNA knockdown in A549 cells. The cells were infected with the lentiviruses and shRNA-expressing cells selected under puromycin pressure, and the HAX-1 knockdown cell lines (shRNA1 and shRNA2) were confirmed, in comparison to the cells with the scramble shRNA (scramble) or cells not transduced (−), with a real-time RT-PCR for HAX-1 mRNA transcript levels. Expression of HAX-1 in knockdown cells was also shown in a western blot analysis. (b–d) Decreased influenza virus replication in HAX-1 knockdown cells. HAX-1 knockdown and control A549 cells were infected with the H9N2 virus at MOI of 0.01, 0.1, or 1.0, and the cultural medium was titrated in MDCK cells with a plaque assay. The titration was performed with duplicates, and the mean titers were exhibited.

To examine the role of HAX-1 in influenza viral infection, we generated HAX-1 knockdown A549 cell line (HAX-1_kd_). As shown in Figure 3(a), HAX-1 was significantly reduced in the HAX-1_kd_ cell line comparing to the A549 cell line infected with a lentiviral vector expressing scramble shRNA (HAX-1_wt_) and normal A549 cells. Specifically, the mRNA of HAX-1 decreased by 75% in the HAX-1_kd_ cells than that in the HAX-1_wt_ cells. Between the two HAX-1_kd_ cell lines, we picked up the one obtained with shRNA2 with higher knockdown efficiency for subsequent experiments.

Next, we examined H9N2 viral replication at different MOI ranging from 0.01 to 1.0, in both HAX-1_wt_ and HAX-1_kd_ cells. Cultural media were harvested at varying time points of 12, 24, 36, and 48 hrs p.i. and subjected to plaque assay. As shown in Figures 3(b)–3(d), lower infectious viral titers were detected in HAX-1_kd_ cells, evidently in the cells with all infectious doses, and in particular when infected at the MOI of 0.01. This result indicates that HAX-1 is essential and may play an important role in promoting AIV H9N2 virus replication in human lung epithelial cells.

### 3.4. N-terminus of PB1-F2 Interacts with HAX-1 in the Mitochondria

**Figure 4:**
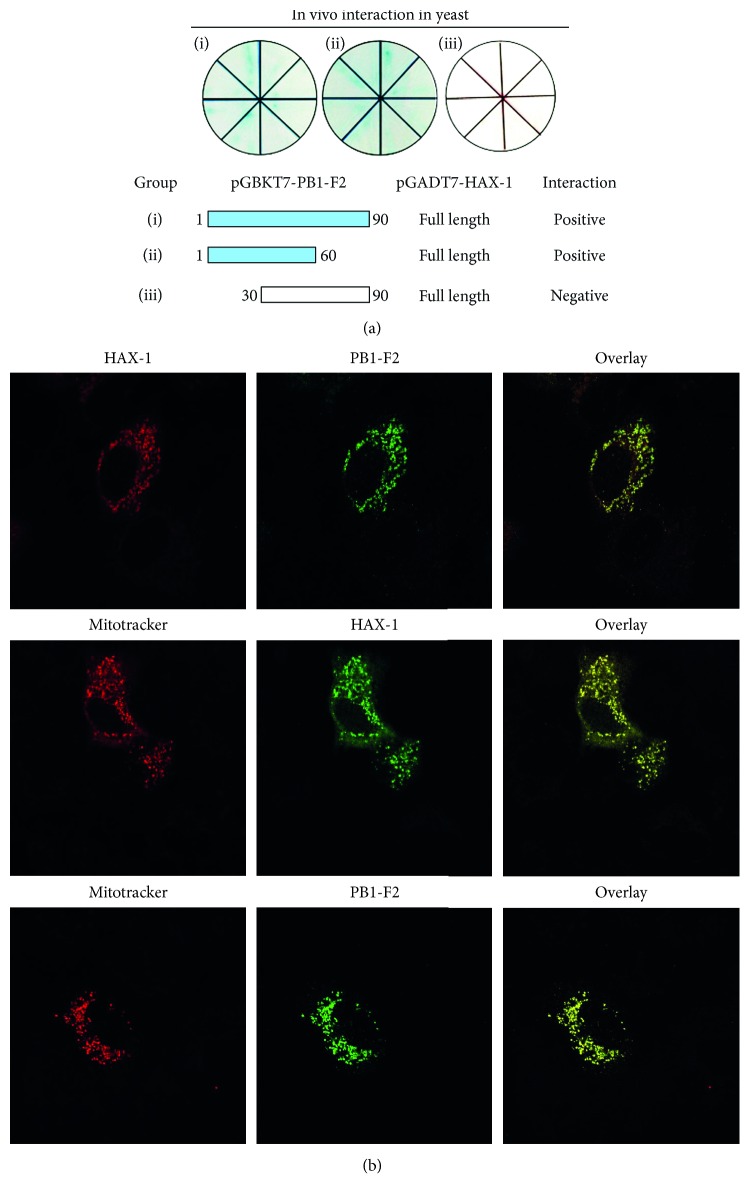
The N-terminus of PB1-F2 was critical for its interaction with HAX-1. (a) Interaction of PB1-F2 and HAX-1 in yeast cells. Yeast cells were cotransformed with pGBKT7-PB1-F2 expressing a full length of 1–90 residues and a segment of the N-terminal 1–60 residues or C-terminal 30–90 residues, together with pGADT7-HAX-1 (full length). One week later, growing clones on each SD/dropout plates were propagated and validated on X-*α*-Gal/SD/dropout plates. Clones generating blue indicate positive interaction, whereas white color indicates negative interaction. (b) Colocalization of PB1-F2 and HAX-1 in the mitochondria of A549 cells. A549 cells were transfected with empty vectors (not shown) or transfected with plasmids expressing HAX-1 or/and PB1-F2 for 24 hrs, subjected to immunofluorescence staining with respective antibodies. The cells were costained with MitoTracker (middle and bottom panels).

In order to biochemically identify the residues in PB1-F2 interacting with HAX-1, we generated truncated fragments of PB1-F2. These fragments included one containing only the N-terminal 60 amino acids, PB1-F2ΔC30, and another with the deletion of the first 29 amino acids at the N-terminus, PB1-F2ΔN29. In cotransformed yeasts with the truncated PB1-F2 fragments and HAX-1, PB1-F2ΔC30 was able to interact with HAX-1, but PB1-F2ΔN29 failed (Figure 4(a)), suggesting that the N-terminal 29 amino acids of PB1-F2 were involved in its interaction with HAX-1. Since the mitochondrial targeting sequence (MTS) of PB1-F2 is an amphipathic *α*-helix at the carboxyl terminus [27], the interaction of PB1-F2 at its N-terminus with HAX-1 would not interfere with its trafficking and translocation to the mitochondria during viral infection.

To evaluate whether PB1-F2 of the H9N2 virus is colocalized with HAX-1 physically in the cell, we cotransfected A549 cells with pcDNA3.1-Flag-PB1-F2 and pcDNA3.1-HA-HAX-1. At 24 hrs after transfection, both HAX-1 and PB1-F2 were distributed in the cytoplasm and appeared to have similar and overlapping distribution, indicating that HAX-1 and PB1-F2 are colocalized (Figure 4(b)). Furthermore, our data showed that both HAX-1 and PB1-F2 are colocalized with the mitochondria as shown by Mitotracker in cotransfected cells, suggesting that HAX-1 and PB1-F2 are most likely localized in the mitochondria (Figure 4(b)).

### 3.5. HAX-1 Attenuated the Caspase-9 Activity Induced by PB1-F2

**Figure 5:**
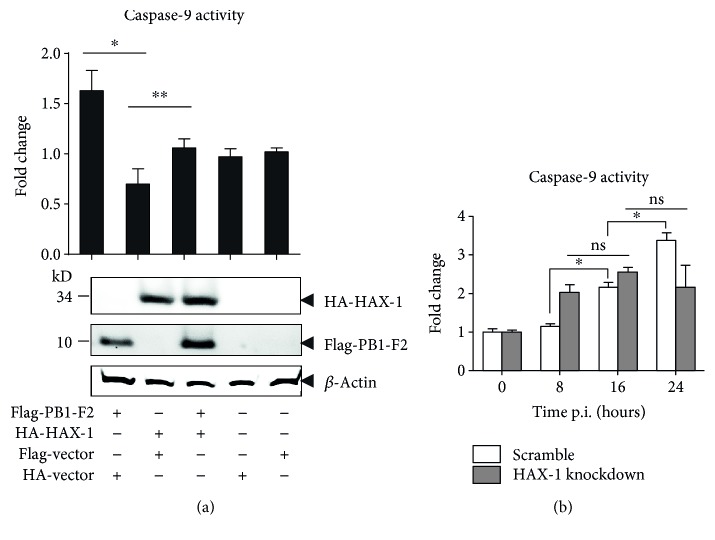
Attenuation of PB1-F2-induced caspase-9 activation by HAX-1. (a) Caspase-9 activity was suppressed by HAX-1 in transfected lung epithelial cells. A549 cells were transfected with plasmids expressing either PB1-F2, HAX-1, or both, and enzymatic activities of caspase-9 in the cell lysates were determined by incubating the lysates with the substrate LEHD-pNA at 37°C for 2 hrs for colorimetric development. The caspase activities were calculated and expressed as relative fold change against the control transfected with a blank plasmid. Expression levels of HAX-1 and PB1-F2 were shown in western blot analysis. (b) Suppression of activated caspase-9 in HAX-1 knockdown cells infected with the H9N2 virus. HAX-1 knockdown or scramble control cells were infected with H9N2 (wt) viruses. Enzymatic activities of caspase-9 in the cell lysates were determined by incubating the lysates with the substrate LEHD-pNA for colorimetric development. Triplicates were performed, and the values of mean ± SD were shown (Student's *t*-test, ^∗^*p* ≤ 0.01 and ^∗∗^*p* ≤ 0.05; ns: no significance).

To investigate the biological significance of the HAX-1 and PB1-F2 interaction, we transfected A549 cells with plasmids expressing either HAX-1, PB1-F2, or both, and caspase-9 enzymatic activities were measured in the lysates prepared from the transfected cells at 16 hrs post transfection. Expression of PB1-F2 alone enhanced the caspase-9 activity, while HAX-1 appeared to have little effect on the activity when expressed alone. However, when the cells were cotransfected with both, the increased caspase-9 activities by PB1-F2 were reduced, indicating that HAX-1 attenuated proapoptotic activity of PB1-F2 in the lung epithelial cells (Figure 5(a)).

Next, we examined the caspase-9 activity in HAX-1_wt_ cells or HAX-1_kd_ cells when infected with H9N2 virus. The caspase-9 activity increased significantly in HAX-1_kd_ cells at 8 hrs p.i. but higher caspase-9 activity was observed in HAX-1_wt_ cells at 24 hrs p.i. (Figure 5(b)). These data indicate that apoptosis was activated more vigorously in the absence of HAX-1, likely induced by PB1-F2, at the early stage of infection. As a result, fewer viruses were produced, leading to decreased apoptosis at the later stage of infection (24 hrs p.i.).

### 3.6. Effect of HAX-1 Deficiency on Apoptosis in Infected HAX-1_kd_ Cells

**Figure 6:**
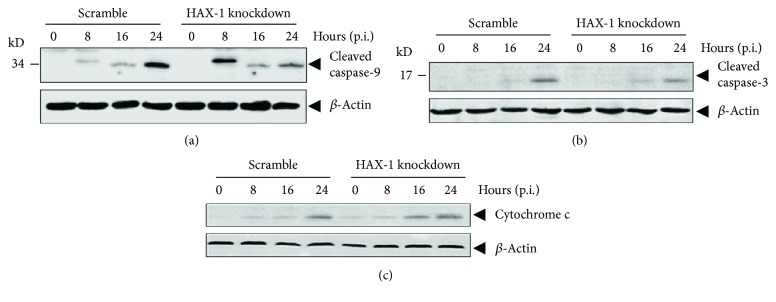
Early cleavage and activation of caspase-9 in HAX-1 knockdown lung epithelial cells infected with the H9N2 virus. Increased caspase cleavage and activation at the early stage of infection in HAX-1 knockdown cells. HAX-1 knockdown (HAX-1_kd_) and control (scramble) (HAX-1_wt_) cells were infected with the H9N2 virus, and the total cell lysates were analyzed by western blot analyses with anti-cleaved caspase-9 (a) and caspase-3 (b) antibodies. The cytosolic fraction of the lysate was also subjected to western blot analysis with an anti-cytochrome c antibody (c).

As shown in Figure 5(b), the activities of caspase-9 in HAX-1_kd_ cells were higher than those in HAX-1_wt_ cells at 8 hrs p.i. induced by H9N2 infection, which indicates a stronger activation of apoptosis in infected cells. To evaluate the activation of apoptosis without HAX-1, we infected HAX-1_kd_ and HAX-1_wt_ cells with H9N2 (wt) virus at an MOI of 1 and analyzed the cleavage status of caspase-9 and caspase-3 during the course of infection. As shown in Figures 6(a) and 6(b), significantly more cleaved/activated caspase-9 (p37 subunit cleaved at Asp330) was produced in HAX-1_kd_ cells compared to that in HAX-1_wt_ cells at 8 hrs p.i. and more cleaved caspase-3 appeared to be present at 16 hrs p.i. in HAX-1_kd_ cells. However, the activation of caspases appeared to be opposite at the late stage of infection: at 24 hrs p.i., a lower amount of cleaved caspase-9 (p35 subunit cleaved at Asp315) and cleaved caspase-3 was observed in HAX-1_kd_ cells than in HAX-1_wt_ cells. Moreover, cytochrome c release into the cytosol increased in HAX-1_kd_ cells compared to that in HAX-1_wt_, indicating that increased caspase-9 activation may be related to a higher level of cytochrome c in the cytosol in the absence of HAX-1 (Figure 6(c)).

## 4. Discussion

It remains unclear whether PB1-F2 is a virulence factor and, if so, what accounts for the mechanism in influenza viral infection. In this study, we investigated the underlying mechanism by which PB1-F2-induced apoptosis is regulated in AIV H9N2 virus-infected human lung epithelial cells. PB1-F2 is a nonstructural protein encoded by many influenza virus strains [6]. It was considered to be a mitochondrial protein promoting apoptosis in infected immune cells [5]. In HEK293T and A549 cells, PB1-F2 colocalizes and binds mitochondrial antiviral signaling protein (MAVS) via its C-terminus and thereby inhibits MAVS-mediated type I interferon synthesis and decreases mitochondrial membrane potential [28, 29]. Furthermore, PB1-F2 binds other mitochondrial components, including channel Tom40, nucleotide-binding oligomerization domain-like receptor X1 (NLRX1) and P3 (NLRP3), and impairs host innate immunity [30–33]. PB1-F2 induced apoptosis by activating procaspase-9 activation as shown in this study, which was originally identified in immune cells. PB1-F2-activated apoptosis may be inhibitory to influenza virus replication since cell death that occurs in the early stage of infection would shorten host cell survival, resulting in decreased viral replication. Thus, we postulated that PB1-F2-induced apoptosis could be regulated by an unknown mechanism in influenza virus-infected cells. In this study, we found that PB1-F2 interacted with HAX-1, which may suppress its activation of mitochondria-mediated procaspase-9 activations.

Using a PB1-F2-deficient H9N2 virus in this study, we found that the viral replication was inhibited, demonstrating that PB1-F2 is critical to viral replication in lung epithelial cells, which may be caused by its importance to viral RNA polymerase activity [34]. Studies have also indicated that PB1-F2 was implicated in suppressing early induction of interferons, contributing to viral virulence and pathogenesis *in vivo* [35, 36], although PB1-F2 could, in contrast, exacerbate interferon induction as an opposite of NS1 in infected lung epithelial cells [37].

HAX-1 is known to associate with hematopoietic lineage cell-specific protein 1 (HCLS-1), a substrate of Src family tyrosine kinases (Suzuki et al., 1997). HAX-1 exists in many tissues of mice and humans [38], was first observed to interact with HCLS-1, and is involved in B cell development [39, 40]. HAX-1 participates in antiapoptosis by inhibiting procaspase-9 processes [41]. The interaction of HAX-1 and PB1-F2 may provide a novel mechanism for PB1-F2 to be regulated by a cellular protein in H9N2 AIV-infected lung epithelial cells. Since HAX-1 is suppressive to apoptosis through binding to procaspase-9, an association of HAX-1 and PB1-F2 during the infection may sequester PB1-F2 from activating procaspase-9, resulting in decreased activation of caspase-9. This hypothesis is supported by our observation that the caspase-9 activity induced by PB1-F2 can be attenuated by the overexpression of HAX-1 in cotransfected cells (Figure 5). We also showed that when HAX-1 was reduced in shRNA knockdown lung epithelial cells, the activation of procaspase-9 was initiated earlier at 8 hrs post infection, which, however, was only achieved much later (at 24 hrs) in normal cells (Figure 6). This result suggests that HAX-1 plays a role in the regulation of apoptosis at the early stage of infection in the presence of PB1-F2. Our study further confirmed that HAX-1 is critical to AIV H9N2 replication in human lung epithelial cells. In HAX-1 knockdown lung epithelial cells, the virus replicated at lower titers than in normal cells (Figure 3), indicating that HAX-1 facilitates influenza virus replication. Likely, the presence of HAX-1 could maintain the cells viable with a delayed apoptotic process, which may be beneficial to viral replication. When HAX-1 was reduced in the knockdown cells, much earlier initiation of procaspase-9 activations (at 8 hrs p.i.) would promote cell death prematurely and yield fewer progeny viruses (Figures 3 and 6).

Our study indicates that HAX-1 may play a beneficial role in the regulation of influenza virus replication in the lung epithelial cells. HAX-1 has two Bcl-2 homolog domains and was originally considered to function through its association to the inner mitochondrial membrane rhomboid protease PARL to proteolytically activate Omi/HtrA2, an antiapoptotic serine protease, and eliminate active proapoptotic Bax from the outer membrane [42]. This was not, however, observed in human lung epithelial cells in this study. We therefore propose that HAX-1 may play a beneficial role in virus replication, prolonged cell survival, and inhibiting apoptosis. This function may depend on cell type or specific virus subtype. A recent report showed that HAX-1 binds to PA, a subunit of the influenza viral RNA polymerase complex, and impedes its nuclear translocation and viral replication [43]. Although the PA sequences are conserved between the H1N1 (A/WSN/1933) [43] and the H9N2 viruses used in this study, there are only 59% of sequence identity in PB1-F2, and PB1-F2 of different subtype influenza viruses may not equally interact with HAX-1. Association of HAX-1 with either PA or PB1-F2 or both could be virus strain or subtype-specific, which may cause distinct consequences.

Although PB1-F2 has been reported to be a promoting factor for apoptosis in infected cells [5, 12, 18], it was considered unable to induce apoptosis in epithelial cells in earlier studies [5]. We found, however, that PB1-F2 of the H9N2 virus could increase caspase-9 activity (Figure 5), and apoptosis was decreased when the cells were infected with mutant H9N2 (ΔPB1-F2) virus at least at the early stage (Figure 1(d)) in human lung epithelial cells. Indeed, influenza viruses are variable in their sequences and structures, and many influenza virus strains only possess a truncated and dysfunctional PB1-F2, among which pandemic (2009) H1N1 virus is exemplary. Variable PB1-F2 functions may be observed in different viral infections. In fact, PB1-F2 is even dispensable to some viral strains for viral virulence and pathogenesis, including many H1N1 viruses.

In summary, we have found that in human lung epithelial cells, PB1-F2 of an H9N2 AIV is critical to viral replication but can also induce apoptosis. PB1-F2-induced apoptosis, which may be inhibitory to viral replication, is regulated by HAX-1, a host protein, resulting in decreased procaspase-9 activations and apoptosis. HAX-1 facilitates influenza virus replication in the lung epithelial cells probably through a mechanism including suppressing apoptosis and promoting Bax expression (data not shown). PB1-F2 may be a virulence factor but also dispensable depending on influenza virus strains. Comparative studies of PB1-F2 from different viruses in various tissue types and species are warranted to further elucidate its functions and precise roles in viral pathogenesis, both in avian species and in humans.

## Figures and Tables

**Table 1:** PB1-F2 interacting proteins identified by the yeast two-hybrid system.

Gene ID	Symbol	Official full name
8409	UXT	Ubiquitously expressed prefoldin-like chaperone
150684	COMMD1	Copper metabolism domain-containing 1
1278	COL1A2	Collagen type I alpha 2
2335	FN1	Fibronectin 1
4522	MTHFD1	Methylenetetrahydrofolate dehydrogenase 1
5193	PEX12	Peroxisomal biogenesis factor 12
57231	SNX14	Sorting nexin 14
10572	SIVA1	SIVA1 apoptosis-inducing factor
10742	RAI2	Retinoic acid-induced 2
22902	RUFY3	RUN and FYVE domain-containing 3
10456	HAX1	HCLS1-associated protein X-1
3492	IGH	Immunoglobulin heavy locus
